# Postharvest Management of Fruits and Vegetables—Series II

**DOI:** 10.3390/foods13071049

**Published:** 2024-03-29

**Authors:** Yoshihiro Imahori, Jinhe Bai

**Affiliations:** 1 Graduate School of Agricultural, Osaka Metropolitan University, Osaka 599-8531, Japan; 2 Horticultural Research Laboratory (USDA-ARS), Fort Pierce, FL 34945, USA

Fruits and vegetables are crucial nutritional sources of carbohydrates, protein, minerals, vitamins, and dietary fiber, offering significant benefits to human health. Their high water content, however, makes them highly susceptible to dehydration and mechanical damage after harvest. Being living organisms, these horticultural crops undergo continuous physiological and biochemical changes, leading to extensive losses due to microbial effects, physical injury, and postharvest senescence. These losses significantly impact the quality and quantity of fruits and vegetables, affecting the balance between harvest and consumption. Effective postharvest management is therefore essential for maintaining quality and extending shelf life, thereby reducing economic losses.

This Special Issue compiles a collection of recent research in the field, featuring ten scientific papers, including eight research articles and two reviews, covering various aspects of postharvest management.

## 1. Ripening, Ethylene, and the Application of 1-Methylcyclopropene (1-MCP)

Rapid ripening significantly contributes to the swift deterioration of climacteric fruits post-harvest, a common occurrence in apples, pears, mangoes, and papayas. Ethylene is a crucial factor in this process, playing a pivotal role in promoting both respiration and ripening [1]. The chemical compound 1-methylcyclopropene (1-MCP) effectively binds to the ethylene receptors in plants, acting as a competitive inhibitor [2]. This binding mechanism helps mitigate the influence of ethylene, consequently reducing the loss of quality in fresh produce [2,3].

The application of 1-MCP, both before and after harvest, has achieved considerable success in various fruits, particularly in apples [3,4] and pears [3]. Recent efforts have expanded its use to fruits typically consumed at a ripe stage, such as European pears, mangoes, papayas, bananas, tomatoes, and kiwifruit [5]. In cases such as that of apples, where ripening is not required before consumption, it is crucial to maintain 1-MCP usage within the approved dosage limit of 1 ppm or lower [6]. For other fruits, the 1-MCP dosage must be carefully balanced; it should be high enough to extend storage life, yet low enough to allow for the fruit to ripen after storage, optionally with additional ripening treatments [5].

Research by Bai et al. [7] and Shu [8] has shown the importance of skillfully manipulating 1-MCP application for optimal results. Strategies such as delaying the application to stages of partial ripening [8] and reducing the dosage through a combined approach [7] have proven effective. Dias et al. [5] have further compiled the most effective methods for reversing the effects of 1-MCP, contributing valuable insights to the postharvest management of climacteric fruits.

Among the contributions in this issue, three articles involve the application of 1-MCP. Yuan et al. examined the treatment of 1-MCP with or without combination with melatonin on mangoes, observing improved visual quality and increased activities of antioxidant enzymes. Prange and Wright reviewed the additional effects of 1-MCP on apples and pears stored in controlled atmosphere (CA) conditions, finding that some cultivars can be stored at higher temperatures, potentially leading to energy savings and quality benefits. Shah et al. reviewed advances in loquat storage technology, showing that 1-MCP treatment alleviates chilling injury (CI) in loquat, inhibits reactive oxygen species (ROS) production, and maintains membrane integrity, thereby reducing internal browning and the incidence of fruit decay.

## 2. Controlled and Modified Atmosphere

CA is most commonly used on apples and pears, employing a combination of reduced oxygen and increased carbon dioxide, along with cold temperature and high relative humidity (RH), to reduce water loss, lower respiration rate, prevent decay development, and extend storage life [6]. In comparison with CA, modified atmosphere (MA) is used for film packaging in small-scale and short-term storage, making it more suitable for retailers. The atmosphere composition in MA packaging (MAP) is less precise. Bai et al. [9] demonstrated a perforated packaging which substantially enhanced the quality of small fruit in modified humidity packaging without considerable changes in the oxygen and carbon dioxide.

In this Special Issue, three articles relate to CA/MA. Sinde et al. investigated the effects of passive and active MA on the quality of fresh-cut melons, indicating the importance of packaging design for different melon types to avoid fermented off-flavor associated with ethanol and ethyl acetate. Shah et al. summarized the applications of controlled and modified atmospheres in loquat storage, emphasizing the specific oxygen concentration needs for different fruits. Compared to the low oxygen concentration of 1–3% in apples and pears [6], the required concentration for loquat is much higher, at 10–13%. Prange and Wright intensively examined the effects of CA on apples and pears. A dynamic controlled atmosphere (DCA) was introduced to indicate the ways in which to adjust the oxygen and carbon dioxide concentration during storage to fit the changing plant physiological status in fruit.

## 3. Natural Wax and Edible Coating Application

Natural wax on the fruit surface plays a key role in protecting against water loss from the fruit and partially creates low-oxygen and high-carbon dioxide conditions within the fruit [10]. Under modern postharvest handling systems, washing and brushing remove most of the natural wax cover, making the fruit more susceptible to water loss and reducing the effectiveness of CA. Edible coatings function similarly to natural wax but offer enhanced effects in terms of water protection and internal gas control [10].

Two articles in this Special Issue relate to natural wax and edible coatings. Bai et al. stored winter melons at 20 °C for four months. The natural wax was initially thin directly after harvest; however, its thickness increased gradually during storage. The nutritional and phytonutritional contents, along with the volatile profile, were well-maintained after four months of storage. The appearance remained good even after six months of storage at 20 °C (unpublished data). Obenland et al. explored the performance of a variety of coatings and found that the coatings did not effectively limit weight loss through either the cuticle or stem end of the blueberries. On the contrary, the application of coatings caused a loss of bloom on the blueberry surface, which negatively affected the appearance and marketability. Although fruits are covered with natural wax, the postharvest application of edible coatings is a standard procedure for many of them. This is carried out to reduce water loss, inhibit respiration, slow down postharvest deterioration, and enhance the appearance of apples, pears, citrus fruits, nectarines, and peaches [10].

## 4. Cold Storage and Chilling Injury

Cold storage is the most important technology in fresh fruit and vegetable storage. Many fruits originating from tropical and subtropical regions, as well as some from temperate regions, such as mangoes, grapefruits, winter melons, tomatoes, loquats, and even some apple varieties, are susceptible to chilling injury (CI). CI in fresh fruits refers to damage caused by exposing the fruit to low, but not freezing, temperatures. The symptoms vary depending on the fruit type, but commonly observed signs include surface pitting, internal browning, water soaked appearance (translucency), abnormal ripening, off-flavors and odors, and increased susceptibility to decay [11].

Many fresh products discussed in this Special Issue face CI challenges, including loquats, melons, winter melons, and apples. Cold storage is used for the quality management of loquat fruit; however, the susceptibility of some cultivars to CI can lead to browning and other disorders. Although many melon cultivars are chilling-sensitive, fresh-cut melons do not exhibit clear CI phenomena. Winter melons are susceptible to CI when stored below 13 °C but retain a long storage life at room temperature. Prange and Wright reviewed updated storage temperature recommendations and the factors influencing these temperatures for apples and pears, based on the accumulated postharvest data from the last 20 years. Apple cultivars have been divided into two storage temperature groups (0 to 1 °C and >1 °C) based on chilling sensitivity. Gradual cooling, rather than rapid cooling, is recommended for apple cultivars, especially for chilling-sensitive cultivars. European pear cultivars are held at storage temperatures close to or just below 0 °C since they are not chilling-sensitive, and most cultivars require a cold temperature to induce ethylene production and ripening, especially if picked early for long-term storage. Asian pears apparently have higher-temperature requirements in CA, compared with European pears. The temperature recommendations for regular air (RA) and CA storage differ in some apple and European pear cultivars. The CA recommendation is, on average, approximately 0.9 °C higher for apple cultivars and approximately 0.5 °C higher for pear cultivars, compared with RA. Research evidence suggests that some apple and pear cultivars can be stored at higher temperatures in DCA than in CA, leading to possible energy savings and quality benefits.

## 5. Decay Control

Fresh produce contains 80–95% of water and rich nutrients, which is an ideal medium for infection and the growth of plant pathogens. In this issue, two articles focus on fruit postharvest decay. Gong et al. examined metabolic changes in apples during *Penicillium expansum* infection, offering insights into decay control strategies. Olmedo et al. explored the efficacy of thymol vapors in managing grapefruit diseases, demonstrating effectiveness and safety.

The application of low-residue chemicals or natural compounds in active packaging systems has been intensively researched for controlling postharvest food safety and decay. Sun et al. [12,13,14] used controlled-release chlorine dioxide and carvacrol to control microbial growth. Miranda et al. [15] applied coatings incorporated with ginger oil to preserve papaya postharvest quality and prevent decay in papaya.

## 6. Molecular Markers

The integration of Genome-Wide Association Studies (GWAS) [16], quantitative trait loci (QTL) [17], and single-nucleotide polymorphism (SNP)-based DNA markers [18] represents a cutting-edge approach in the genetic improvement of fruits and vegetables. The analyses are powerful tools used in the agricultural sector, particularly in the improvement of fruit and vegetable crops. GWAS involves scanning the genomes of many different plants to identify genetic variants associated with desirable traits, such as chilling injury tolerance, increased flavor quality, or disease resistance. In contrast, QTL analysis focuses on pinpointing specific regions in the genome (loci) that are responsible for quantifiable traits such as disease tolerance. SNP-based DNA markers are a crucial component in this process. These markers are variations at a single DNA base pair, and they serve as precise genetic signposts. They are used to track the inheritance of specific traits and facilitate marker-assisted selection in breeding programs. By combining GWAS, QTL, and SNP markers, researchers and breeders can more efficiently identify and select for genes that confer advantageous characteristics, leading to the development of fruit and vegetable varieties that are better-suited to various environmental conditions, such as chilling temperatures [19] and plant pathogen pressure [20], and consumer preferences, such as certain volatile profiles [21,22]. This triad of genomic tools has significantly accelerated the pace of crop improvement, enabling more targeted and effective breeding strategies. In this Special Issue, Dos-Santos et al. investigated the effect of QTLs on the volatile compound composition in melons, showing the potential of molecular markers in crop improvement.

In summary, this Special Issue provides a comprehensive exploration of methods and advancements in preserving the quality and extending the shelf life of fruits and vegetables post-harvest. It underscores the critical roles of ripening management, atmospheric conditions, temperature control, and molecular genetics in crop improvement and postharvest quality. This collection of articles offers a detailed overview of the current and emerging postharvest management techniques, highlighting their essential role in maintaining the nutritional and economic value of fruits and vegetables.
